# The Management of Wrist Fractures During COVID-19: A Preliminary Report

**DOI:** 10.7759/cureus.19982

**Published:** 2021-11-29

**Authors:** Isaac C Okereke, Omar Ramadan, Sridhar R Sampalli

**Affiliations:** 1 Trauma and Orthopaedics, The Royal London Hospital, London, GBR; 2 Trauma and Orthopaedics, Queen Mary University of London, London, GBR; 3 Trauma and Orthopaedics, University Hospitals Dorset NHS Foundation Trust, Poole, GBR; 4 Trauma and Orthopaedics, Salisbury District Hospital, Salisbury, GBR

**Keywords:** fragility fractures, fracture-clinic, non-operative, covid-19, wrist fractures, virtual follow-up

## Abstract

Background

Due to the current COVID-19 pandemic, there has been an increase in the need for the virtual follow-up of patients. These innovations in clinical care have helped to reduce hospital attendance of patients and the spread of the virus. Injuries such as wrist fractures that are non-obligatory are increasingly being followed up virtually. This paper compares the early experience of management of wrist fractures in a District General Hospital in the United Kingdom during the COVID-19 pandemic lockdown with a similar period before.

Methods

A retrospective study of the management and clinical follow-up of all skeletally mature patients seen in the Accident and Emergency (A&E) department with a radiologically confirmed distal radius fracture after imposition of COVID-19 lockdown measures in the United Kingdom (between March 23, 2020 and May 24, 2020), and comparison with a control group of patients with distal radius fractures seen within a similar time frame the previous year (March 23, 2019 to May 24, 2019).

Results

During the COVID-19 lockdown, a total of 39 skeletally mature patients (85% females; average age of 70.4* *years [SD: 14.6]) who had suffered a wrist fracture were seen. A total of 23% of the patients had surgical fixation. The others were managed conservatively and followed up regularly in the fracture clinic and by virtual telephone consultation in 15% (p > 0.05) compared to the previous year. Three patients who had an AO/OTA Type-C fracture were not keen on surgery, citing the COVID-19 pandemic. Patients had their operations at 5.2 days post-injury on average compared to the pre-COVID average of 6.4 days post-injury.

Conclusion

This preliminary study showed that patients considered "high risk" (as per the UK government guidance on shielding and protecting people who are clinically extremely vulnerable from COVID-19) with low functional demands who had suffered fractures of the distal radius were followed up mostly virtually after their first A&E attendance, thereby eliminating unnecessary hospital attendances. There was no difference in the epidemiology of wrist fractures pre- and post-COVID-19 lockdown. No COVID-positive patients were treated.

The limitations of this study are the fact that it is cross-sectional with a lack of patient-reported outcome measures (PROM). As this was only a preliminary study to assess initial results, it will be followed up by a full report assessing outcomes at defined intervals.

## Introduction

The WHO confirmed a novel strain of COVID-19 was responsible for the outbreak of a respiratory-type illness in Wuhan City, Hubei province of China, and declared this a pandemic on March 12, 2020 [1]. With an R rate (R0) of 2.0-3.0, a death rate of approximately 5%, and no immunologic memory in humans [2], the COVID-19 pandemic strained the capacity of healthcare systems around the world in ways that have not been witnessed in recent history. To flatten the pandemic curve, the UK government imposed a total lockdown on March 23, 2020, banning all “non-essential” travel and contact with people outside one’s home, and shutting almost all schools, businesses, venues, facilities, amenities, and places of worship. The mortality rate in the United Kingdom at the time of the imposition of lockdown measures was 335, with a total of 6,650 people testing positive. As of October 25, 2021, the confirmed COVID-19 cases in the United Kingdom, according to Public Health England (PHE), was 8,773,674 with mortality of 162,620 [3], making the United Kingdom the second-worst hit after Belgium in deaths per capita from the COVID-19 infection.

The NHS England (NHSE), in preparing for the first wave of COVID-19 viral infection in the population, declared a Level 4 National Incident on the January 30, 2020. Taking a cue from the massive burden, the pandemic had put on the Italian healthcare system and anticipating the immense pressures the NHS resources would come under at the peak of the epidemic in the United Kingdom, NHS Trusts were directed to “redirect staff and resources” to free up the maximum possible in-patient and critical care capacity, as well as, prepare for and respond to the projected large numbers of COVID-19 patients anticipated to require respiratory support [4]. All non-urgent elective surgical operations were postponed from the April 15, 2020 at the latest for at least three months. New government rules required that people went into self-isolation for up to two weeks on contact with a symptomatic patient and for seven days if they developed any of the viral disease symptoms. The effect of the COVID-19 pandemic took its toll on the healthcare workforce. This was previously uncharted territory, requiring new guidelines to quickly be drawn up for managing scarcely available surgical workforce and theatre resources.

While acknowledging the requirement for the surgical specialities to adapt to these new changes in healthcare delivery, the four Royal Colleges of surgery in the United Kingdom released guidance for surgeons working during the COVID-19 pandemic. At the centre of these new guidelines was the necessity for surgeons to triage and deliver healthcare to patients for maximal benefits as in a mass casualty scenario, as well as protect and preserve the surgical workforce [5]. As with mass casualty incidents (MCI), standard practices must be modified to minimise infection or death of sub-specialised staff to preserve the ability to face surgical emergencies and associated activities that will continue to occur or perhaps increase during MCI [6]. The British Orthopaedic Association (BOA) and the NHS England (NHSE) also released guidelines that encouraged surgeons to “balance optimum treatment of a patient’s injury or condition against clinical safety and resources” [7, 8].

Considering the restrictions on available NHS resources caused by the COVID-19 pandemic, some fractures which would have been managed by surgical fixation have been treated conservatively. Wrist fractures are an example of traumatic injuries that have seen a leaning towards non-operative treatment in this period. Fragility fractures of the wrist are a common Accident and Emergency (A&E) presentation, most of which can be managed in the interim by manipulation under a block and application of a plaster cast. A number of these injuries may be unstable and require surgical fixation. The best surgical management of these fractures remains controversial. The results of the distal radius acute fracture fixation trial (DRAFFT) showed no difference in functional outcomes between patients who had suffered a fracture of the distal radius that were either managed with a volar locking plate or K-wires [9]. It is standard practice to follow-up these patients in a fracture clinic post-injury to assess stability and function, with most patients requiring at least one hospital follow-up visit. The UK government’s guidance on shielding and protecting people, who are clinically extremely vulnerable from COVID-19, strongly advised people with the following medical conditions to shield themselves; an organ transplant, cancers, severe respiratory diseases, rare diseases and inborn errors of metabolism, immunosuppression therapies, and pregnant women [10].

In this paper, we compared the management of wrist fractures in skeletally mature adults at a District General Hospital in the periods before and during the COVID-19 lockdown.

## Materials and methods

Study population

A retrospective study of all skeletally mature patients seen in the A&E department with a radiologically confirmed wrist fracture between the imposition of lockdown measure in the United Kingdom on March 23, 2020 and May 24, 2020, and a control group of patients with the same injury seen in the same period the previous year (March 23, 2019 and May 24, 2019). Clinical records using specific Systemized Nomenclature of Medicine (SNOMED) codes were requested from the Medical Records Department and retrieved from the Institutional database. Data collected included patient demographics, date of attendance, and diagnosis in A&E. X-rays were reviewed using the Picture Archiving Communications System (PACS) and wrist fractures classified using the AO/OTA classification system. In addition, the Trust’s Lorenzo software for patient records was interrogated and reviewed for A&E notes, fracture clinic attendances and letters, and Virtual Fracture Clinic (VFC) letters.

Wrist fracture management pathway 

**Figure 1:**
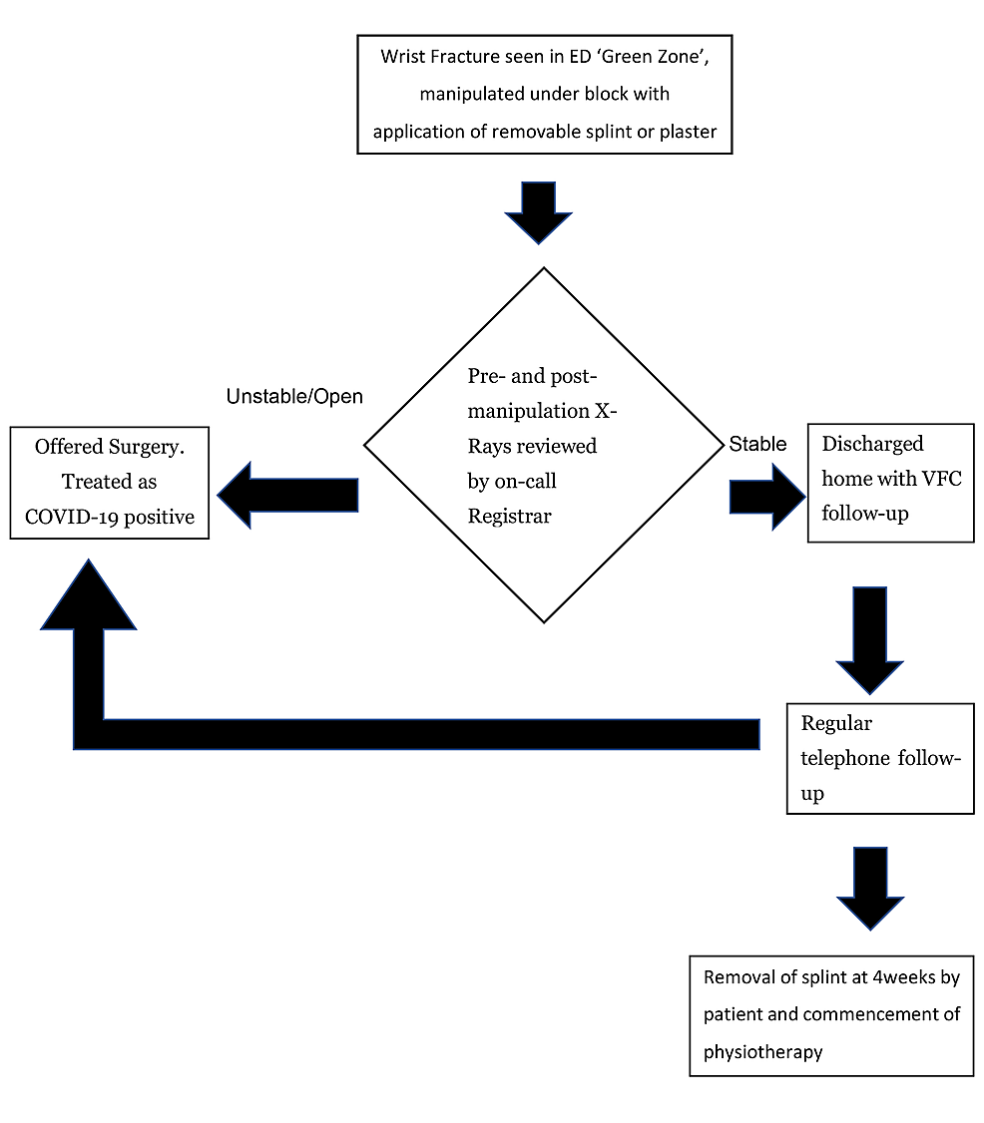
Flow chart for management of wrist fractures.

Patients with a displaced wrist fracture and no symptoms of COVID-19 are seen in the minor injuries section of the A&E department by Emergency Nurse Practitioners (ENPs), which is designated a “Green Zone” (Figure 1). The Green Zone is kept “Green” by allowing the review of only patients with no clinical symptoms of COVID-19 or a positive swab test at triage in this area. Manipulation for displaced fractures is done under a haematoma block with Entonox and immobilised in a removable plaster or splint. The pre and post-manipulation X-rays were reviewed remotely by the on-call Orthopaedics Registrar. Fractures with a volar displacement which are considered inherently unstable, are considered for surgical intervention and variables such as age, demand, hand-dominance, and co-morbidities determine candidates for surgery. Patients with a stable fracture configuration or an acceptable reduction are discharged home and followed up within two days on average in the VFC. The VFC which had been in operation at our facility for at least two years before the COVID pandemic is a consultant-led virtual clinic where radiographic images of all referred cases are reviewed alongside clinical records and a definitive management plan formulated. The idea is that all stable injuries seen in ED and discharged home are referred through this pathway. If a distal radius fracture is deemed not sufficiently reduced on review at the VFC, a fracture-clinic appointment is arranged for a re-manipulation and check radiographs, or surgical fixation is offered to the patient. Distal radius fractures whose reductions are considered satisfactory are followed-up by regular telephone consultations to avoid travel inconveniences, maintain social distancing, and reduce viral transmission [11]. During telephone follow-up, particular attention is paid to reported pain levels, the presence of swelling, and neurological changes in the affected wrist. At about four-to-six weeks post-injury, patients are encouraged to remove the wrist plaster or splint and commence hand physiotherapy. Patients who undergo surgery are immobilised in removable splints, discharged on the same day, and have absorbable sutures used for wound closure to avoid the need for further hospital visits.

## Results

Between March 23, 2019 and May 24, 2019, a total of 56 adult patients were seen in the A&E with wrist fractures. A total of 75% were females, and the average age at time of injury was 63.67years (SD 21.4). 30% had surgical fixation of their fractures by either K-wires or plates and screws. Two of the patients with AO/OTA Type-C fractures declined surgery and were managed conservatively, one of them having presented four weeks post-injury after being on holidays. The average day of operation was 6.4 days post-injury, and the average age of patients who had surgery was 55 years (Table 1).

**Table 1:** Patient demographics, type of wrist fracture sustained using the AO/OTA classification, management, time to surgery, and follow-up pre- and post-lockdown. **: Fisher’s exact test; *: Independent student’s t-test; N/A: Not applicable.

Demographic	Pre COVID-19 lockdown	Post COVID-19 lockdown	Difference/Odds ratio (95% CI)	P-value
	n = 56	n = 39		
Mean age, years (SD)	63.67 (21.4)	70.4 (14.6)	Diff 6.73 [-1.1, 14.5]	0.091*
Sex, n%				
Female	42 (75%)	33 (85%)	Reference	
Male	14(25%)	6(15%)	OR 0.6 [0.2, 1.6]	0.3
AO/OTA classification, n%				
Class A	15 (27%)	7 (18%)	Reference	
Class B	28 (50%)	28 (72%)	OR 2.14 [0.76, 6.1]	0.2
Class C	13 (23%)	4 (10%)	OR 0.7 [0.2, 2.8]	0.5
Management, n%				
K-Wires	0	2 (5%)		
Locking plate	17 (30%)	6 (15%)	Reference	
External fixation	0	1 (3%)		
Non-operative	39 (70%)	30 (77%)	OR 1.5 [0.6, 3.7]	0.43
Follow-up, n%				
Fracture Clinic	56 (100%)	33 (85%)	N/A	
Telephone	0	6 (15%)	N/A	0.04
Mean time to surgery, days (SD)				
	6.4 (4.2)	5.2 (3.5)	Diff 1.2 [-2.2, 4.6]	0.22*
Age at surgery, years (SD)				
	55 (19.4)	62.3 (17.0)	Diff 7.3 [-7.14, 21.7]	0.2*

A total of 39 patients were seen in A&E with a wrist fracture between March 23, 2020 and May 24, 2020. There were 85% females with an average age of 70.4 years (SD: 14.6). A total of 23% of the patients had surgical fixation. The others were managed conservatively and followed up regularly by telephone consultation or, if required, physically reviewed in fracture clinic. Three patients who had an AO/OTA Type-C fracture declined surgery, citing the COVID-19 pandemic. Patients were operated averagely on day 5.2 post-injury. A total of 15% of patients were followed up virtually and, in most cases, did not need further hospital assessment after discharge from A&E. This is clinically significant when compared to the previous year, where every patient (except one lost to follow-up) was physically followed-up in the fracture clinic (p < 0.05, Fisher’s exact test). 

## Discussion

The COVID-19 pandemic is a significant challenge to global healthcare systems. With over 240 million confirmed cases and more than four million mortalities globally [11, 12], healthcare systems have suffered strains on capacity and workforce depletion from many ill people requiring intensive care [13]. The increased risk to orthopaedic surgeons and theatre staff has been well documented by Hirschmann MT et al. [14].

Surgical specialities have had to revisit guidelines for operative management of patients due to a reduction in workforce and available hospital resources. While obligatory fractures such as frailty fractures of the hip have continued to be managed operatively as routine to avoid complications associated with recumbence and immobility, the management of non-obligatory fractures like distal radial fractures has been approached more conservatively [11, 14]. The British Orthopaedic Association Audit Standards for Trauma (BOAST) management of distal radial fractures standards published in 2017 recommends consideration of non-operative management in patients older than 65 years with a dorsally displaced distal radius fracture and with no significant deformity or neurological compromise [15].

For patients whose fractures are amenable to non-operative management in plaster or splint, there is now a reliance on telephone follow-up clinics to avoid hospital visits that are not necessary. This is with a view for more thoughtful utilisation and preservation of scarce resources to deal with a potential future wave of the COVID-19 pandemic. Even though telemedicine has been around for some time, the COVID-19 pandemic has led to the widespread adoption of this technology across different subspecialties in patient care. Telemedicine refers to the use of communications networks for delivering health services by care providers. Instead of transporting the patient to the clinical specialist through telemetry, we can transmit the specialist's knowledge to the patient. Telemedicine lends itself especially in underserved communities where there is a shortage or absence of adequate clinical care, such as in remote areas. It is practical and sustainable in many clinical areas, and in some cases, more cost-effective than traditional face-to-face consultations [16-18]. 

Although there was no statistically significant difference in the epidemiology of wrist fractures following COVID-19 at our centre, the lockdown measures implemented, such as quarantines, bans on travel, and self-isolation for those with higher risks, has led to reduced levels of physical activity, especially in the elderly population, thus predisposing to loss of muscle mass and function and contributing to osteoporosis. It may well be that the impact of the COVID-19 in terms of fragility fractures is yet to be felt.

Telemetric consultations have apparent limitations, such as the inability to carry out clinical examinations and the absence of visual cues while communicating with patients. Whilst physical examination is an integral aspect of orthopaedic assessments, especially for elderly patients who often have hearing difficulties or suffer dementia, thus making virtual consultations tedious, these challenges can however be overcome with more detailed documentation, shared decision making, as well as actively involving the general practitioners and care homes in virtual consultation outcomes [19]. Elimination of modifiable risk factors for osteoporosis such as smoking, alcohol abuse and physical inactivity should also be considered during telemetric follow-up consultations.

Most patients managed conservatively and with virtual follow-ups in this study (n = 6/39; 15%) commenced physiotherapy at about five weeks after initial injury. One patient developed a malunion.

The care pathway for the management of wrist fractures at our facility was adapted to protect and preserve the surgical workforce, reduce hospital stay of patients, and emphasise the closed treatment of fractures, as elaborated by John Charnley in his 1950 monograph [20].

## Conclusions

In our study, patients with a fracture of the wrist who have low functional demands and are considered too high risk for operative management due to the COVID-19 pandemic were managed conservatively irrespective of injury type. In line with new guidelines for the management of non-obligatory fractures, such as distal radius fractures, these patients were treated in plaster or splint and with regular virtual follow-ups. The decision to operate and type of operation was made on a case-by-case basis. In this study, pre and post-COVID-19 data did not show any change in epidemiology or surgical fixation methods for patients with wrist fractures. 

This study is a preliminary study and so is limited by the lack of long-term follow up of patients and PROMs.
